# Almond (*Prunus dulcis* cv. Casteltermini) Skin Confectionery By-Products: New Opportunity for the Development of a Functional Blackberry (*Rubus ulmifolius* Schott) Jam

**DOI:** 10.3390/antiox10081218

**Published:** 2021-07-29

**Authors:** Monica R. Loizzo, Rosa Tundis, Mariarosaria Leporini, Gilda D’Urso, Rossella Gagliano Candela, Tiziana Falco, Sonia Piacente, Maurizio Bruno, Francesco Sottile

**Affiliations:** 1 Department of Pharmacy, Health and Nutritional Sciences, University of Calabria, 87036 Rende, Italy; monica_rosa.loizzo@unical.it (M.R.L.); mariarosarialeporini@tiscali.it (M.L.); tiziana.falco@unical.it (T.F.); 2 Department of Pharmacy, University of Salerno, 84084 Fisciano, Italy; gidurso@unisa.it (G.D.); piacente@unisa.it (S.P.); 3 Department of Biological, Chemical and Pharmaceutical Sciences and Technologies, University of Palermo, Viale delle Scienze, Parco d’Orleans II, 90128 Palermo, Italy; r.gaglianocandela88@gmail.com (R.G.C.); maurizio.bruno@unipa.it (M.B.); 4 Interdepartmental Research Center “Bio-Based Reuse of Waste from Agri-Food Matrices” (RIVIVE), University of Palermo, Viale delle Scienze, Parco d’Orleans II, 90128 Palermo, Italy; francesco.sottile@unipa.it; 5 Department of Architecture, University of Palermo, Viale delle Scienze, Parco d’Orleans II, 90128 Palermo, Italy

**Keywords:** by-products, *Prunus dulcis* cv. Casteltermini skin, *Rubus ulmifolius*, LC-ESI/LTQOrbitrap/MS, GC–MS, carbohydrate hydrolysing enzymes, lipase

## Abstract

This work proposes for the first time a model for reusing almond (*Prunus dulcis* cv. Casteltermini from Sicily, Southern Italy) skin to formulate a new functional blackberry (*Rubus ulmifolius* Schott) jam. For this purpose, blackberries were analysed fresh and as jam, traditionally prepared with a minimum fruit amount of 80%. Different percentages of almond skin (20, 15, and 10% *w*/*w*) were added to jam. The phytochemical profile of enriched jam was investigated by LC-ESI/LTQOrbitrap/MS analyses. Anthocyanins, hydrolysable tannins, and triterpenoids were identified in a blackberry extract, while proanthocyanidins, flavonoids, and oxylipins were identified in an almond extract. The *n*-hexane extract of *P. dulcis* skin, investigated by GC–MS, evidenced linoleic, palmitic, and oleic acids as the main abundant compounds. Samples were investigated for their antioxidant activity using DPPH, ABTS, β-carotene, and FRAP tests. The hypoglycaemic and hypolipidemic effects were studied by α-amylase, α-glucosidase, and lipase inhibitory assays. In order to evaluate the effect of thermal process on enriched jam bioactivity, pasteurisation was applied. An increase in activities for all samples was observed, in particular for jam enriched with 20% *w*/*w* of almond skin. Based on obtained data, and supported by sensory analysis, we propose enriched jam as a promising source of compounds useful for preventing diseases associated with oxidative stress.

## 1. Introduction

During industrial food processing and production, a large amount of waste is generated. These by-products are constantly increasing, and for this reason, in recent decades, attempts to develop reuse and recovery methods for residual food have been proposed [1]. The European Commission is undertaking a series of actions for a shift to a circular economy, in which products and waste are recovered, regenerated, and reused for a new production cycle. Much importance has been given to the recovery of bioactive compounds from different by-products, useful as ingredients for food supplements or active compounds in pharmaceutical products [2].

Recently, the interest in functional food consumption has increased. The functional components of food can be effectively applied in the treatment and prevention of several diseases acting simultaneously at different or identical target sites with potential physiological benefits [3].

*Rubus ulmifolius* Schott (Roseaceae) is a perennial shrub, widely distributed in Europe, North and South America, Asia, and North Africa, characterised by several fleshy drupelets that are black at fully maturity stage [4]. Blackberries are consumed fresh or as derivate products, such as juices, jams, liqueurs, desserts, breakfast cereals, etc., due to their delicious flavour. This species, together with other *Rubus*, is largely investigated for its high content of bioactive compounds, such as polyphenols, fibres, vitamins, and minerals [5]. Due to these compounds, blackberries have been recognised as healthy fruits, and their consumption has been recommended not only as fresh products but also as dietary supplements and in fortified food products [6,7,8]. Solverson et al. [9] conduced a randomised crossover study to investigate the effects of blackberry intake on energy substrate use and gluco-regulation in obese volunteers consuming a high fat diet, and demonstrated that this fruit, consumed at a dose of 600 g per day for one week, significantly decreased fat oxidation and insulin sensitivity compared to the control group.

In the last ten years, the worldwide market demand for the consumption of almond fruit (*Prunus dulcis* (Mill.) D.A.Webb, *Prunus amygdalus* L.) has significantly increased due to the physico-chemical, nutritional, and sensorial features of these fruits [10].

*P. dulcis* cv. Casteltermini (sin. Regina) is a traditional, widely diffused species in Sicily where it represents a significant part of the agricultural sector. It is composed of hundreds of local varieties characterised by a wide genetic variability that also appears evident in the whole fruit [11].

Sicily is the largest Mediterranean island with an area of 25,500 km^2^.

The Sicilian ecosystems contain 3252 vascular floral species, 321 of which are endemic [12]. In the Sicilian province Palermo is found one of the most interesting vascular floral species, with respect to plant biodiversity (60% of whole plant species endemic in Sicily) [13]. Considering that climate change directly affects plant secondary metabolites such as polyphenols, it is important to investigate the chemical pattern of species with different geographical origins [14]. Almond kernels are consumed whole or in slices; blanched, unblanched, or dry-roasted. The main food industry use is bakery and confectionery products [15].

The kernel is obtained by removing the fruit’s envelopes. The fleshy pericarp and mesocarp form the fibrous hull, and the mature stony endocarp forms the light brown shell. Inside the shell, the kernel is surrounded by a light brown coloured and thin tegument called skin. Therefore, there are hard-shelled and semi-hard-shelled varieties which greatly influence the yield of shelled seeds as well as the amount of by-products at shelling. In hard-shelled varieties, the part of waste can also reach 75% of the total fruit weight, of which 90% is shell and 10% skin.

This skin protects the almond from oxidation and microbial contamination, and it is considered an agricultural by-product [16,17]. This by-product is rich in polyphenols, fibres, and lipids. Mandalari et al. [18] reported that Californian almond skin contains about 46.3% of dietary fibre, 3.2% of soluble dietary fibre, 23.2% of lipids, and 11.5% of proteins. Recent studies have indicated that, although it is considered a by-product with very low economic value, it contains about 60.0–80.0% of the total phenolic compounds that exist in the whole fruit [19,20,21,22,23]. Moreover, clinical studies have demonstrated that a regular almond consumption can reduce the risk of several diseases in humans [24]. In part, this bioactivity can be ascribed to the phytochemicals present in the skin, particularly to the polyphenols with proven antioxidant and health-promoting activity [18,21,25].

Based on these healthy properties, in the last decades, several works investigated the possibility to enrich food matrices with polyphenols. Among selected matrices, until now, jam has scarcely been chosen [26,27,28,29]. However, jam is often one of the foods of which consumption is recommended during breakfast, which is considered, from a nutritional point of view, the most important meal. For these reasons, the present research aimed to propose a model for the application of almond (*Prunus dulcis* cv. Casteltermini) skin to enrich blackberry (*Rubus ulmifolius*) jam and to obtain a functional food useful for the prevention and/or treatment of prediabetic conditions with a strong link to the Sicilian territory. For this purpose, the chemical composition, antioxidant, hypoglycaemic, and hypolipidemic activities of blackberries, blackberry jam, almond skin by-product, enriched jam, and pasteurised enriched jam extracts were investigated.

## 2. Materials and Methods

### 2.1. Chemicals and Reagents

The solvents used in this study were obtained from VWR International s.r.l. (Milan, Italy). Delphinidin 3-*O*-glucoside, cyanidin 3-*O*-glucoside, pelargonidin-3-*O*-glucoside, procyanidin B1, kaempferol-3-*O*-glucoside, kaempferol-3-*O*-rutinoside, chlorogenic acid, quercetin-3-*O*-glucoside, quercetin-3-*O*-rhamnoside, quercetin, (+)-catechin, (-) epicatechin, pruning, and rosmarinic acid were used as standards, and the other reagents were purchased from Sigma Aldrich (Milan, Italy).

### 2.2. Samples Preparation

The almonds were supplied by a local farmer and originated from almond trees cv. Casteltermini cultivated in Agrigento (Sicily, Italy). They belong to the group with hard shells, a low shelling rate, and a high quantity of by-products. The skin was separated by water blanching at 95 °C for 3 min followed by peeling rollers. After separation, the skin was immediately dried in a hot-air oven at 60 °C until they reached a constant moisture content. The overall moisture content achieved by the skin samples ranged from 4 to 5%. The almond skin was pulverised using a grinder (Moulinex DJ3001, Moulinette Compact) (Milan, Italy).

The blackberries were collected in August 2019 from plants growing wild in the Alimena area (Sicily, Italy). Blackberry jam was prepared following the Sicilian traditional procedure. First, 500 g of sucrose was added to dried fruits, stirred, and left overnight at 4 °C. Then, the mixture was cooked over low heat and stirred constantly until it thickened. The still-boiling jam was poured into the sterilised jars, leaving about 2 cm of edge space. The jars were closed well with the caps, turned over, and left to cool. In order to obtain anthocyanin fractions, the procedure reported by Rodriguez-Saona et al. [30] was followed. Briefly, blackberries and jam (50 g) were mixed with a solution of acetone/water (350 mL, 70/30 *v*/*v*) and blended for 10 min. The mixture was filtered and washed with 50 mL acetone/water (70/30 *v*/*v*). The resulting solution was extracted with chloroform (400 mL) and the upper aqueous layer (115 mL) was kept in refrigerator.

In order to eliminate other water soluble compounds (sugars, etc.), the aqueous coloured solution was subjected to column-chromatography over silica gel 100 C18-reversed phase (Sigma-Aldrich), first using acidic water (0.01 M in HCl) and then acidic methanol (0.01 M in HCl) as eluents. The methanolic solution was evaporated to give 1.14 g of a solid containing the anthocyanins (yield 2.28% with respect to dried fruits). With an identical procedure, 53.7 g of blackberry jam gave 617 mg of anthocyanins (yield 1.14% with respect to jam).

Blackberry and jam extracts were obtained using ultrasound-assisted maceration with ethanol (EtOH). Three extraction cycles with an ultrasonic frequency of 40 kHz at a temperature of 30 °C for 60 min were conducted for each sample in a water bath 3800-CPXH (Branson, Milan, Italy). After each cycle, the mixture was filtered and the solvent was removed using a rotary vacuum evaporator at 30 °C. Additionally, 500 g of almond skin powder were freeze-dried to give 220 g of dry material. Then, 180 g of dry material was extracted by a Soxhlet extractor (3 h) with *n*-hexane to give, after solvent evaporation, 18.0 g of oil (yield 10.0%). The residual material was successively extracted with EtOH (Soxhlet, 3 h) to give 3.8 g of gum (yield 2.1%).

The almond skin extracts were also obtained using ultrasound-assisted maceration with EtOH and *n*-hexane (3 × 1 h). The almond skin powder was added to blackberry jam to obtain final concentrations of 20, 15, and 10% *w*/*w*.

The enriched jam was stored at 4 °C. In order to evaluate the impact of food processing on the jam bioactivity, the enriched jam was pasteurised [1]. Table 1 reports samples details and abbreviations.

### 2.3. Total Phenol, Flavonoid and Anthocyanin Content

The total phenol content (TPC) was evaluated as previously described using the Folin-Ciocalteu reagent [1]. The results are expressed as mg of chlorogenic acid equivalents (CAE)/g of extract. The determination of total flavonoid content (TFC) was made as previously reported [1]. The results are expressed as mg quercetin equivalents (QE)/g of extract. The differential pH method was used to determine the total anthocyanin content (TAC) [31]. The results are expressed as equivalent mg of cyanidine-3-*O*-glucoside/g of extract.

### 2.4. LC-ESI/LTQOrbitrap/MS/MS^n^ Analysis

The sample extracts were prepared in a concentration of 1 mg/mL (methanol/water) and 5 μL were injected and analysed by LC coupled with an LTQ-Orbitrap FTMS hybrid system (LC-ESI/LTQOrbitrap/MS) in negative and positive ion mode, following a previously published protocol with slight modification [32]. In detail, a quaternary Accela 600 pump and Accela autosampler connected with a LTQ-Orbitrap XL mass spectrometer with electrospray ionisation (ESI) was used for the analysis. The analytical parameters were the following: a Phenomenex Luna C18 (150 × 2.1 mm; 5 µm) column, and a solvent system of H2O + 0.1% formic acid (A) and CH3CN 0.1% formic acid (B).

The gradient program was: 0–30 min, from 5 to 95% (B); then, 5 min to 95% (B) and back to 5% (B) for 5 min. The flow rate was 0.2 mL/min. ESI source parameters in negative ion mode: capillary voltage −48 V; tube lens voltage −176.47 V; capillary temperature 280 °C; sheath and auxiliary gas flow (N_2_) 15 and 5; sweep gas 0; spray voltage 5 V. In positive ion mode: capillary voltage 49 V; tube lens voltage 120 V; capillary temperature 280 °C; sheath and auxiliary gas flow (N_2_), 30 and 5; sweep gas 0; spray voltage 5 V. A full range acquisition covering *m*/*z* 120–1600 was used for both polarities. A fragmentation study was performed using the data dependent scan mode, selecting precursor ions corresponding to the most intense peaks in the LC-MS spectra. Xcalibur software version 2.1 was used for instrument control, data acquisition, and data analysis.

### 2.5. UPLC-ESI-QTrap-MS/MS Analysis by Multiple Reaction Monitoring

Quantification was performed using a Shimadzu Nexera LC system coupled with a Sciex 6500 QTrap MS. Analytical parameters were the following: kinetex EVO C18 column (Phenomenex) (100 × 2.1 mm i.d., 1.7 µm d); mobile phases consisted of H_2_O + 0.1% formic acid (A) and CH_3_CN + 0.1% formic acid (B); flow rate of 0.3 mL/min; gradient program: 0–10 min from 5% to 98% (B); and then back to 5% (B) for 3 min. 5 µL of each sample was injected. The available commercial standards were selected and prepared (1 µg/mL in methanol) for the injection into the ESI source of mass spectrometer, which was operated in positive and negative ion mode.

Specific transitions were optimised by selecting a specific precursor ion and product ion for each standard in order to get the best transitions in the MRM mode. Using the same method, declustering potential (DP), entrance potential (EP), collision energy (CE), and collision cell exit potential (CXP) values were optimised for each standard to obtain the best response. Data were analysed using Analyst 1.6.2 software (ABSciex, Foster City, CA, USA).

The following parameters were used for the ESI source: curtain gas (CUR) = 40; collision gas (CAD) = medium; IonSpray voltage (IS) = −4500; temperature (TEM) = 350 °C; ion source gas 1 (GS1) = 25; ion source gas 2 (GS2) = 25; dwell time for each analyte was 20 ms. This method was validated according to the European Medicine Agency (EMA) guidelines. A standard solution was prepared with a concentration of 0.05, 0.1, 0.25, 0.5, and 1.5 µg/mL in methanol.

### 2.6. Gas Chromatography–Mass Spectrometry (GC–MS) Analysis

Almond skin *n*-hexane extract was subjected to basic trans-methylation using potassium hydroxide in methanol before the Hewlett-Packard gas chromatograph (GC) (Agilent, Milan, Italy) equipped with a non-polar HP-5 capillary column (30 m × 0.25 mm, 0.25 μm), associated with a Hewlett-Packard mass spectrometer (MS) (Agilent, Milan, Italy) [33].

The ionisation of the sample constituents was performed in electronic impact (EI, 70 eV). The analyses were carried out with the following temperature schedule: isotherm at 50 °C for 5 min, temperature increase from 50 to 250 °C at 5 °C/min, and finally isotherm at 250 °C for 10 min. Helium was used as a carrier gas. The compound identification corresponding to methyl esters (FAMEs) was based on the comparison of the mass spectral data with the Wiley 128 library, and referred to the spectral data of a standard mixture of FAMEs. The compounds’ relative concentrations were calculated based on peak areas without using correction factors.

### 2.7. In Vitro Antioxidant Activity

In order to establish the antioxidant activity, a multi-target approach was applied [1]. In the FRAP assay, the capacity of samples to induce the reduction in tripyridyltriazine (TPTZ)-Fe^3+^ was evaluated. The absorbance was read at 595 nm. The 2,2-azino-bis(3-ethylbenzothiazoline-6-sulfonic) acid (ABTS) and 2,2-diphenyl-1-picrylhydrazyl (DPPH) assays were applied to investigate the radical scavenging ability. In the DPPH test, the DPPH radical was made to react with samples, and after 30 min, the bleaching of the radical was defined at 517 nm. In the ABTS assay, an ABTS radical cation solution was mixed with the samples, and after 6 min, the discolouration was measured at 734 nm. The capacity of samples to inhibit the lipid peroxidization was tested using β-carotene bleaching test.

### 2.8. Hypoglycaemic and Hypolipidemic Activity

The inhibition of α-glucosidase and α-amylase was investigated as a potential target for the modulation of post-prandial hyperglycaemia, an important tool in the management of prediabetes conditions [1]. The potential to reduce fat absorption of samples was assessed by a pancreatic lipase inhibitory assay, as previously reported [1]. In this assay, a mixture of samples—4-nitrophenyl octanoate (NPC), Tris-HCl buffer (pH 8.5), and enzyme solution—were added in a 96-well plate and incubated at 37 °C for 30 min. The absorbance was determined at 405 nm.

### 2.9. Sensory Analysis

A sensory analysis was conducted by a selected and trained panel comprising 10 adult judges (5 males and 5 females, aged between 20 and 52 years, recruited from the Department of Pharmacy, Health and Nutritional Sciences, University of Calabria staff).

To evaluate the intensity of olfactory, gustatory, and visive attributes (colour, odour, appearance, aroma, sweetness, acidity, astringency, and mouthfeel), each subject received 17 samples (unidentified, with randomly assigned three-digit codes): control jam samples (without almond skin), not pasteurised (EM) and pasteurised (EMP), and enriched jam samples, not pasteurised (EM1, EM2, and EM3) and pasteurised (EMP1, EMP2, and EMP3).

Samples were served at 12–15 °C in tasting glasses and coded. In the questionnaire presented to the judges, they were requested to observe and taste each coded sample with the provided bread, and grade them based on a 10-point hedonic scale, showing least acceptable to most acceptable in all attributes. They were also provided with potable water to rinse their mouth after evaluating each sample to check taste interference. A quantitative method was performed to define the sensory profile of each sample, and the results were analysed and reported graphically in spider web form using Microsoft Office Excel 2010.

### 2.10. Statistical Analysis

Experiments were carried out in triplicate. Prism GraphPad Prism version 4.0 for Windows (GraphPad Software, San Diego, CA, USA) was used to calculate the concentration, giving 50% inhibition (IC_50_). In the biological tests, differences within and between groups were evaluated by a one-way analysis of variance test (ANOVA) followed by a multi-comparison Dunnett’s test. In the chemical analysis, Tukey’s test was used to determine any significant difference in chemical parameters among the investigated samples.

Pearson’s correlation coefficient (*r*), assessment of repeatability, linear regression, average, and relative standard deviation calculation were completed using Prism GraphPad Prism version 4.0 for Windows. Principal Component Analysis (PCA) was applied by SPSS software for Windows, version 15.0 (Chicago, IL, USA). Statistical analyses were performed using SPSS software for Windows (SPSS Inc., Elgin, IL, USA) 22.0 Version.

## 3. Results and Discussion

### 3.1. Samples Extract

#### 3.1.1. Total Phytochemical Contents

The content of bioactive compounds in blackberries, blackberry jam, and almond skin extracts was investigated (Table S1, Supplementary Materials). The highest TPC was recorded in the hydroalcoholic extract of blackberries (189.31 mg CAE/g of extract), followed by the ethanolic extract of the almond skin (56.68 mg CAE/g of extract). The greatest content of flavonoids was found in the EB sample (30.26 mg QE/g of extract). The AB extract showed the highest TAC (7.52 mg of cyanidine-3-*O*-glucoside/g of extract). Food processing determined a reduction in TAC [34]. Indeed, the TAC in the jam extract was lower than in the blackberry fruit extract, with a value of 2.06 mg of cyanidine-3-*O*-glucoside/g of extract. Previously, Milbury et al. [35] investigated the TPC and TFC in several almond varieties (Butte, Carmel, Fritz, Mission, Monterey, Nonpareil, Padre, and Price) growing in California, and found values ranging from 9.9 to 26.8 mg gallic acid equivalent/100 g for Fritz and Price, respectively. These values are lower than those found in *P. dulcis* cv Castelminiti skin. A higher TPC was found by Mandalari et al. [18], who investigated the amount of bioactive compounds in Californian almond skin powder, natural and after the blanching process, and found TPC values of 2474 and 278.9 mg gallic acid equivalent/100 g, respectively. This evidence confirms that the blanching process reduced the TPC in the matrix. Several factors can affect the TPC in almond skin, including cultivar, environmental and climatic conditions, cultivation practices, processing and storage, as well as extraction methods. All this makes it difficult to compare TPC data [35,36].

Oancea et al. [37] investigated the impact on TPC and TAC of traditional jam processing of different fruits, including blackberries. A reduction in TPC of about 60% was found after jam making, whereas a drastic reduction (~80%) was found for blackberry jam in comparison to fresh fruits. In both cases, the reduction is the consequence of the temperature applied during jam making [38].

#### 3.1.2. LC-ESI/LTQOrbitrap/MS/MS^n^ Analysis

Samples were analysed by LC-ESI/LTQOrbitrap/MS/MS^n^. According to their accurate mass, characteristic fragmentation pattern, retention time, as well as literature data on *R. ulmifolius* and *P. dulcis*, and whenever possible, by comparison with authentic standards, the identity of most of peaks was putatively attributed (Table 2, Table S2).

In particular, a detailed screening of EB and ET highlighted the presence of anthocyanins, hydrolysable tannins, and triterpenoids in EB, and proanthocyanidins, flavonoids, and oxylipins in ET. Some compounds (**16**, **26**–**27**, **32**, **33**, **36**, **39**, and **42**) were detected in both extracts. In particular, compounds **16** and **39** were putatively identified as dihydroquercetin glucoside and isorhamnetin glucoside, respectively, already reported in almond skin [39] but detected in *Rubus* species for the first time. Compounds **27** and **42** were putatively identified as methylellagic acid glucuronide and iperoside respectively, reported in *Rubus* sp. but never in *Prunus* sp.

*R. ulmifolius* fruit ethanol extract (EB) revealed the presence of five anthocyanins (**7**, **11**, **12**, **17**, **21,** and **30**) [40]; compounds **11**, **12** and **30** were identified and confirmed with commercial standards as cyanidin 3-*O*-glucoside, pelargonidin 3-*O*-glucoside, and delphinidin 3-*O*-glucoside, respectively.

Compounds **7**, **17,** and **21** showed, in the LC-ESI/HR/MS spectrum, a principal precursor [M + H]^+^ ion, whose fragmentation pattern allowed to identify the occurrence of sugar units linked to a cyanidin aglycone (*m*/*z* 287.05). Compounds **7**, **17,** and **21** were putatively identified as cyanidin dihexoside, cyanidin xyloside, and cyanidin dioxayl glucoside, already reported in *Rubus* fruit by Primo da Silva et al. [4]. Moreover, in the extract of *Rubus* fruit, compounds belonging to hydrolysable tannins (**4–5**, **8**, **25**, **29**) already reported in *Rubus* sp. were detected and identified [41]; the occurrence of triterpenoid compounds (**46**, **51**, **54**, **56**, **57**, **58** and **59**) was remarkable, detected for the first time in *Rubus* fruit but already reported in the leaves of *Rubus* sp. [42]; in addition, flavonols (**10**, **18**, **33**, **34**, **38** and **53**) and a flavanol (**20**) were detected in EB.

The following compounds occurred only in the *P. dulcis* skin extract (ET). In particular, compound **13** showed a parent ion [M-H]^−^ at *m*/*z* 456.1498, thus suggesting the presence of an odd number of nitrogen atoms; the fragmentation spectrum showed a base peak [M-H-133]^−^ at *m*/*z* 323 due to the neutral loss of the mandelonitrile moiety, along with a product ion [M-H-193]^−^ at *m*/*z* 263 and a fragment ion [M-H-235]^−^ at *m*/*z* 221, both related to the cleavage of the cross-ring bonds of glucose 2; moreover, a fragment ion [M-H-277]^−^ at *m*/*z* 179 was observed, and it was related to the glucose moiety. The molecular formula was established as C_20_H_27_O_11_N, suitable with the identity of compound **13** as amygdalin.

Flavanol monomers, such as catechin and epicatechin, along with their oligomers (proanthocyanidins) (**9**, **14–15**, **19**, **22–24**, **28**) already reported in almond skin by Bottone et al. [43] could be detected. Compounds **14** and **22** showed superimposable fragmentation patterns with a base peak [M-H-44]^−^ at *m*/*z* 245, originating from the neutral loss of an acetaldehyde molecule as a consequence of a retro Diels–Alder reaction at the C-ring; the molecular formulae were established as C_15_H_14_O_6_, suggesting two isomers. Due to the intrinsic impossibility to discriminate among regioisomeric forms by LC-MS^n^ experiments, to achieve an unambiguous identification, the Rt of the detected compounds were compared with standard solutions analysed in the same experimental conditions, allowing the identification of compound **14** as (+)-catechin and compound **22** as (-)-epicatechin [44].

Compounds **9** and **19** exhibited superimposable fragmentation patterns with a base peak [M-H-152]^−^ at *m*/*z* 425 originating from a retro Diels–Alder reaction, with the consequent loss of the B-ring; moreover, a diagnostic product ion [M-H-288]^−^ at *m*/*z* 289 suggested two (epi)catechins linked by a B-type linkage. The molecular formula was established as C_30_H_26_O_12_, suitable with the identity putatively attributed to compounds **9** and **19** as B-type (epi)catechin dimers [45].

Furthermore, compounds **15** and **23** showed in MS/MS spectra a base peak [M-H-152–18]^−^ at *m*/*z* 695, generated by the contemporary loss of a water molecule and the B-ring due to a retro Diels–Alder reaction, as well as two diagnostic fragment ions [M-H-290]^−^ at *m*/*z* 575 and [M-H-288]^−^ at *m*/*z* 577, generated by the loss of (epi)catechin terminal and extension units, respectively, both linked to a central (epi)catechin by a B-type linkage; the molecular formulae were established as C_45_H_38_O_18_ [45]. Compound **28** showed a precursor ion [M-H]^−^ at *m*/*z* 575.1182, while in the MS/MS spectrum it exhibited a base peak [M-H-126]^−^ at *m*/*z* 449, generated by the heterocyclic A-ring fission, together with a fragment ion [M-H-286]^−^ at *m*/*z* 289, due to the quinone methide fission of the A-type linkage; the molecular formula was established as C_30_H_24_O_12_, supporting the putative identification of compound **28** as A-type (epi)catechin dimer [45]. Compound **24**, exhibiting in HRMS a parent ion [M-H]^−^ at *m*/*z* 863.1806, showed in the MS/MS spectrum two informative product ions [M-H-288]^−^ at *m*/*z* 575 and [M-H-290]^−^ at *m*/*z* 573, generated by the quinone methide fissions of the linkages of an (epi)catechin extension and terminal units, respectively, linked to the a central (epi)catechin moiety by a B-type and an A-type linkage, respectively; the molecular formula was established as C_45_H_36_O_18_, in accordance with the identity putatively attributed to compound **24** as (epi)catechin-B-(epi)catechin-A-(epi)catechin.

Phenolic compounds **37**, **47,** and **48** were identified as the stilbene glucoside icariside E5, rosmarinic acid, and piceid, respectively, already reported in almond skin by Bottone et al. [43]. Flavanones (**31** and **40**) and flavonol (**35**) were also detected; moreover, it is to be noted that the occurrence of compounds **50** and **55**, whose molecular formulae and fragmentation patterns were similar to two oxylipins already reported by D’Urso et al. [46] in *A. esculentus*, is here detected and putatively identified in *P. dulcis* for the first time.

Oxylipins are hydroxyl fatty acids, which differ from each other by unsaturation degree and number of hydroxyl groups. Product ions generated by one or more consecutive neutral losses of 18 Da allowed us to ascertain the number of hydroxyl groups occurring in the oxylipin structure. Moreover, along with the molecular formula and the ring double bond equivalent value (RDB), the fragmentation pattern allowed, in some cases, to establish the double bond position. By these considerations, compounds **50** and **55** could be putatively defined, as reported in Table 2.

#### 3.1.3. UPLC-ESI-QTrap-MS/MS Quantitative Analysis

After the identification of metabolites in blackberry (EB) and almond skin (ET) extracts, all the samples (EB, ET, EM, AB, and AM) have been analysed by LC-ESI/LTQOrbitrap/MS/MS^n^, confirming the presence of the compounds listed in Table 3.

To evaluate the content of the main phenolic compounds detected by LC-ESI/LTQOrbitrap/MS in EB, ET, EM, AB, and AM extracts, a selective and sensitive method for quantitative analysis was developed by UPLC-ESI-QTrap-MS/MS in multiple reaction monitoring (MRM) mode. MRM is a tandem mass spectrometric technique in which a specific transition from a precursor ion to a product ion is monitored for each analyte.

In detail, an electrospray ionisation is used, followed by two stages of mass selection: a first stage (MS1), in which the mass of the intact analyte (parent ion) is selected and, after fragmentation of the parent ion by collision with gas atoms, a second stage (MS2), in which a specific fragment of the parent ion is selected, collectively generating a selected reaction monitoring (plural MRM) assay. In this way, only the selected transitions are monitored [47].

In detail, anthocyanins **11–12** and **30**, flavonols **32–33,** and prunin (**40**), in the MS/MS spectrum were characterised by the loss of 162 Da corresponding to glucose unit, originating from an intense peak of their corresponding aglycone. Anthocyanins **17** and **21** in the MS/MS spectrum showed a main product ion at *m*/*z* 287 due to the loss of 132 and 306, corresponding to xylose and to dioxayl-glucose units, respectively. MS/MS spectra of flavonols **26** and **36** showed the loss of 146 Da, corresponding to a rhamnose unit generating an intense fragment ion corresponding to their aglycon.

(+)-Catechin (**14**) and (-)-epicatechin (**22**) have the same fragmentation pattern with an intense product ion at *m*/*z* 109; thus, they could be quantified on the basis of their different retention times [47]. Compound **19** showed a diagnostic product ion at *m*/*z* 289, corresponding to the loss of a catechin unit. In addition, the MS/MS fragmentation pattern of rosmarinic acid (**47**) showed a main fragment ion [(M-180-H_2_O)-H]^−^ at *m*/*z* 161, due to the loss of a caffeic acid unit and a water molecule, while the MS/MS fragmentation of chlorogenic acid (**6**) showed the loss of caffeic acid moiety with a main fragment ion at *m*/*z* 191, corresponding to quinic acid. Based on these fragmentations, specific transitions were selected for each compound. Table 3 shows the results obtained for selected metabolites.

The results are reported as average values of three analyses, and expressed as µg of metabolite/g dried extract (DW). In particular, five anthocyanins were quantified, with cyanidin 3-*O*-glucoside (**11**), pelargonidin 3-*O*-glucoside (**12**), and delphinidin 3-*O*-glucoside (**30**) as the most abundant compounds in EB and AB; their levels were lower in the jam extract (EM), in accordance with the results of TAC reported above.

Cyanidin dioxayl glucoside could be quantified only in EB and AB, as it was not detectable in jam samples. Flavanols, like (+)-catechin (**14**), proanthocyanidin B (**19**) and (-)-epicatechin (**22**), together with rosmarinic acid (**47**), and prunin (**40**) were detected and quantified in ET.

#### 3.1.4. Almond Skin n-Hexane Extract GC–MS Analysis

The GC–MS analysis identified the presence of 11 fatty acids (Table 4). The most abundant compound was linoleic acid (63.19%), followed by palmitic acid (16.67%), and oleic acid (8.41%). The almond skin also contained interesting levels of stearic (4.10%) and palmitoleic acids (3.40%).

Previously, only Saura-Calixto et al. [48] had analysed the fatty acids profile of a mixture almond skin from almond cultivar growing in Palma de Mallorca (Spain). Oleic (47.13%), linoleic (40.34%), and palmitic acids (7.53%) were found as the most abundant compounds. Significant differences were found in the essential fatty acid linoleic acid content, which was 1.6-fold higher in the Sicilian almond cv.

Randomised clinical trials have shown that replacing saturated fat with linoleic acid reduces total and LDL cholesterol with a positive effect on coronary heart disease prevention [49]. There is also some in vivo evidence that linoleic acid improves insulin sensitivity in cases of metabolic syndrome or type 2 diabetes [50].

#### 3.1.5. Antioxidant Activity

Blackberry anthocyanin extracts demonstrated the highest activity in DPPH and ABTS tests, with IC_50_ of 1.48 and 0.21 µg/mL, respectively (Table 5). These extracts possessed a greater activity than ascorbic acid. Similar activity was observed with the anthocyanin extracts of blackberry jam. Interesting were also the results obtained from the ethanolic almond skin (IC_50_ of 2.54 µg/mL in ABTS test).

In the FRAP test, the most active samples were the anthocyanin extracts (102.39 and 92.71 μM Fe (II)/g, respectively, for AB and AM). Both extracts exhibited a higher activity than the positive control BHT (63.2 μM Fe (II)/g). The same observation can be made of the blackberry ethanolic extract, which showed a significant and higher activity (87.47 μM Fe (II)/g) compared to the positive control.

The highest antioxidant activity in the β-carotene bleaching test was recorded for blackberry anthocyanin extracts (IC_50_ of 2.15 and 3.92 μg/mL after 30 and 60 min of incubation, respectively). The anthocyanin extract of blackberry jam exhibited similar values of 3.34 and 4.51 μg/mL, after 30 and 60 min of incubation, respectively. Pearson’s correlation coefficient evidenced *r* value of 0.69 for both TAC and FRAP test, and TFC and β-carotene bleaching test.

#### 3.1.6. Hypoglycaemic and Hypolipidemic Effects

Several studies have demonstrated a correlation between oxidative stress, obesity, and type 2 diabetes [1]. For this reason, extracts were tested for their potential inhibitory activity against α-amylase, α-glucosidase, and pancreatic lipase (Table 6). The inhibition of α-amylase and α-glucosidase delayed the digestion with a consequent reduction in the post-prandial glycaemic peak. In both assays, the anthocyanin extracts showed an activity higher than acarbose. Indeed, the AB extract exhibited IC_50_ of 17.59 and 12.77 µg/mL for α-amylase and α-glucosidase, respectively. Similar values were observed for AM with an IC_50_ of 28.42 and 15.75 µg/mL, for α-amylase and α-glucosidase, respectively. Better results were obtained against α-glucosidase, with IC_50_ values in the range 32–78 mg/mL for aqueous extracts, and in the range 28–75 mg/mL for methanolic extracts.

Pancreatic lipase is a key enzyme involved in the digestion of lipids: in the gastrointestinal tract, it breaks down triglycerides into absorbable free fatty acids. Therefore, its inhibition results in a reduction in the absorption of ingested fats with a hypolipidemic effect. As shown in Table 6, the highest inhibitory activity was observed for the AB extract with an IC_50_ of 23.37 µg/mL, followed by the AM extract (IC_50_ of 30.16 µg/mL). Both extracts were more active than the positive control. Pearson’s correlation coefficient demonstrated a positive correlation between TPC, TFC, and the α-amylase, α-glucosidase, and lipase tests. The highest correlations were found between TFC and α-amylase (*r* = 0.94), TFC and α-glucosidase (*r* = 0.62), and TFC and lipase (*r* = 0.73).

### 3.2. Enriched Jam

Based on the obtained results, blackberry jam was chosen as a matrix to be enriched with almond by-product for the development of a functional food. The skin was added to the jam in different concentrations: 20, 15, and 10% (*w*/*w*). Previously, Castelminiti almond skin showed the presence of cellulose, hemicellulose, and lignin with percentages of 38.5, 28.8, and 29.5%, respectively [51]. These fibres may contribute to the functional properties of the investigated jam.

#### 3.2.1. Total Phytochemical Contents

TPC and TFC content was investigated in all enriched samples (Figure S1). As expected, the jam enriched with almond skin showed an increase in the total phytochemicals content, as compared to the control jam. 

The sample enriched with 20% of almond skin (*w*/*w*) exhibited the highest TPC and TFC content (26.96 and 24.12 mg/g, respectively) followed by the sample enriched with 15% of almond skin (*w*/*w*) (22.77 and 22.39 mg/g, respectively). The results also showed that the EMT1 had an increase of +6% in both TPC and TFC compared to the control jam. In EMT2, an increase of +2 and 3% compared to EM was observed respectively for TPC and TFC. A lower value of about 1% was found for EMT3.

The literature studies demonstrated that the addition of plant-derived polyphenols may improve the jam’s quality by increasing their polyphenol content and extending their shelf life [52]. Banaś et al. [27] reported that the addition of plant ingredients (15% chokeberry, 15% elderberry, 8% Japanese quince, 3% flax seed, and 3% wheat germ) to gooseberry jams resulted in an increase in the identified polyphenols. Similarly, the addition of black chokeberry caused an increase in the level of polyphenols by an average of 213%, elderberry by 75%, and Japanese quince by 40%, compared with the strawberry jam without enrichment [28]. Nawirska-Olszańska et al. [29] mentioned the beneficial effect of adding Japanese quince to pumpkin jams on the level of polyphenols. Previously, Wojdyło et al. [26] found an increase in polyphenols after the addition of chokeberry to strawberry jams.

#### 3.2.2. Antioxidant Activity

Table 7 reports the results of the antioxidant activity of the enriched blackberry jam. The increase in antioxidant activity was proportional to the added concentration of almond skin. Indeed, the antioxidant potential was, significantly, highest in the enriched jam at 20% of almond skin (*w*/*w*), with an increase of +20 and +21% in DPPH and ABTS, respectively, compared to the non-enriched jam extract (EM).

The EMT2 increased the radical scavenging activity of +14 and 10% in DPPH and ABTS tests, respectively, compared to the control. A lesser increase was observed for the sample EMT3 (+6 and 7% in DPPH and ABTS tests, respectively).

Similarly, the addition of almond skin (at 10 g/100 g and 20 g/100 g) to a wheat flour basis for biscuit preparation indicated an increase in antioxidant potential [53].

A greater increase in the iron reduction capacity was observed for EMT1, with an 8% increase compared to EM. In the β-carotene test, the increase in antioxidant activity was, significantly, highest for EMT1 compared to EM, with +26% after 30 min of incubation. Similar results were observed for EMT2 (+19 and 17% after 30 and 60 min of incubation, respectively). The EMT3 sample also showed an increase of +17 and 16%, after 30 and 60 min, respectively. The most significant Pearson’s correlation was found between the TFC and FRAP test (*r* = 0.99). TPC had a greater positive correlation with ABTS test (*r* = 0.98).

#### 3.2.3. Hypoglycaemic and Hypolipidemic Activity

The potential of the enriched jam to inhibit α-amylase, α-glycosidase, and pancreatic lipase is reported in Table 8.

The best results were obtained with EMT1, with an inhibition rate of 91, 103, and 96.74% for α-amylase, α-glycosidase, and lipase, respectively. Indeed, this sample determined an increase in the inhibitory activity of +18, 21, and 26% against α-amylase, α-glycosidase, and lipase, respectively.

Interesting results were also observed for EMT2, with an increase in inhibitory activity of +14% for α-amylase, +10% for α-glucosidase, and +21% for lipase enzymes, compared to EM. For EMT3, an increase of +12 and 18% was observed for α-amylase and lipase, respectively, compared to EM. Pearson’s correlation coefficient demonstrated a positive correlation between TPC, TFC, and the α-amylase, α-glucosidase, and lipase tests. The highest correlation was found between TPC and the α-glucosidase test with *r* = 0.99.

### 3.3. Pasteurised Enriched Jam

Enriched and non-enriched blackberry jams were subjected to pasteurisation treatment in order to evaluate the impact of the process on the phytochemicals content and bioactivity.

#### 3.3.1. Total Phytochemical Content

As expected, the thermal process caused a decrease in the phytochemical content. An increase was nevertheless observed in the enriched jam. All enriched jams possessed a higher TPC and TFC content than the pasteurised jam without enrichment. TPC values of 18.48, 17.59, and 17.22 mg/g were observed respectively for EMTP1, EMTP2, and EMTP3. In addition, the following order of TFC values was found: EMTP1 > EMTP2 > EMTP3.

EMTP1 showed an increase of +8 and 5% for TPC and TFC, respectively. An increase in EMTP2 of +7 and 4% was observed for TPC and TFC, respectively, compared to EMP. Similar values were reported for EMTP3.

#### 3.3.2. Antioxidant Activity of Pasteurised Enriched Jam

Table 7 showed the results of the antioxidant activity of enriched jam after pasteurisation process. After pasteurisation, a decrease of 12, 7, 10, 12, and 11% was observed for the DPPH, ABTS, β-carotene bleaching (30 and 60 min of incubation), and FRAP tests, respectively. Despite this decrease, all enriched jams exhibited a greater antioxidant activity than the negative control. In particular, an increase of +21 and 13% for DPPH and ABTS test, respectively, was observed for EMTP1. Of interest is the increase obtained for EMTP2, with +14 and 11%, respectively, for DPPH and ABTS tests. The best activity of EMTP1 was confirmed in the β-carotene test, with an increase of +28 and 27% after 30 and 60 min of incubation, respectively. Additionally, EMTP2 showed an increase of +21 and 20% after 30 and 60 min of incubation, respectively. Pearson’s correlation analysis showed that TPC was positively correlated with the ABTS, FRAP, and β-carotene bleaching tests after 30 and 60 min of incubation with values of *r* = 0.94, 0.97, 0.99, and 0.98 respectively. TFC was positively correlated with the DPPH test (*r* = 0.99).

#### 3.3.3. Hypoglycaemic and Hypolipidemic Effect of Pasteurised Enriched Jam

Table 8 reports the hypoglycaemic and hypolipemic data of enriched and pasteurised jam compared to non-pasteurised enriched jam. Pasteurisation caused a decrease in the inhibition activity of enzymes of 8, 10, and 9%, for α-amylase, α-glycosidase, and lipase, respectively. Despite the decrease, the inhibition potential increased proportionally with the concentration of almond skin added to the jam. The inhibition percentage of α-amylase and α-glucosidase was greater for EMTP1, with an increase of +19 and 18%, respectively. Similar results were obtained for EMTP2, with an increase of +15% against both enzymes. An increase of +11 and 9% was observed of EMTP3, for α-amylase and α-glucosidase, respectively. Regarding pancreatic lipase, an increase was observed in the inhibition percentage, ranging from 17% for EMTP3 to 23% for EMTP1. The Pearson’s coefficient demonstrated a positive correlation between TPC, TFC, and the α-amylase, α-glucosidase, and lipase tests. In all tests, the highest correlations were found in TPC with *r* values of 0.99, 0.97, and 0.97 for α-amylase, α-glucosidase, and lipase, respectively.

#### 3.3.4. Sensory Evaluation of Functional Jam

The sensory analysis is the main aspect for designing new foods suh as our enriched jam. The changes in texture or colour or aroma compared to the original matrix can significantly affect the consumer’s acceptance. Results of sensory evaluation of pasteurised enriched jam are reported in Table S3 and Figure S2. The following sensorial notes were measured: appearance, colour, odour, aroma, sweetness, acidity, and mouthfeel. Generally, the tasting panel did not find any defects in enriched jam. No statistically significant difference was evidenced by the panellists with regard to appearance, colour, or acidity between the enriched jam and the control jam, independent of the percentage of almond skin added. This aspect is very important, since the vision is the only factor that the consumer uses as a basis for the action of purchase [54]. For both odour and aroma, the pasteurised enriched jam was more attractive than EMP, with a maximum score for sample EMTP1 of 8.42 and 8.56, respectively. A statistically significant decrease in sweetness and mouthfeel notes was observed in all enriched jams, in comparison with the control jam. Despite Lauro et al. [25] reported that almond skins possess “woody” and “earthy” organoleptic characteristics that can reduce the consumer’s preferences. In our case, we have not encountered this problem, since these sensory attributes are probably masked by the flavour and taste of the jam.

## 4. Conclusions

In summary, this study aimed to propose the use of almond skin by-products from Casteltermini cv. to formulate a new functional blackberry jam. The jam, enriched with almond skin at 20% *w*/*w*, characterised by the higher content of bioactive compounds, including dietary fibres (cellulose, hemicellulose, and lignin) and polyphenols (such as kaempferol 3-*O*-rutinoside, kaempferol 3-*O*-glucoside, and cyanidin 3-*O*-glucoside), showed the greatest antioxidant effect independently by the applied tests and enzyme inhibitory activities against α-amylase and α-glucosidase, involved in carbohydrate digestion, as well as on pancreatic lipase, with an increased bioactivity of around +20%. The pasteurisation process, necessary for potential commercialisation, although expected, did not negatively affect the bioactivity of this functionalised product, and did not negatively affect its sensory attributes. This aspect appears very important because a product, while being healthy, must always remain pleasant to the consumer’s palate. Moreover, the strong territorial character conferred by the Casteltermini cv., which is typical of Sicily (Italy), could represent a further strength for this new product.

Overall, our results demonstrated that almond skin enriched blackberry jam could be consumed by subjects at a higher risk of getting obesity-associated diseases such as type 2 diabetes.

## Figures and Tables

**Table 1:** Abbreviations used for investigated almond skin, blackberry fruits and jam, and enriched jam extracts.

Sample	Extract	Abbreviation
Blackberry fruits		
	EtOH	EB
	Anthocyanin	AB
Blackberry jam (M)		
	EtOH	EM
	Anthocyanin	AM
Almond skin (T)		
	EtOH	ET
	*n*-hexane	ES
Enriched blackberry jam		
M + 20% T *w*/*w*	EtOH	EMT1
M + 15% T *w*/*w*	EtOH	EMT2
M + 10% T *w*/*w*	EtOH	EMT3
Pasteurised enriched blackberry jam		
M + 20% T *w*/*w*	EtOH	EMTP1
M + 15% T *w*/*w*	EtOH	EMTP2
M + 10% T *w*/*w*	EtOH	EMTP3

**Table 2:** Secondary metabolites identified in ethanol extract of blackberry fruits (EB) and in ethanol extract of almond skin (ET) by LC-ESI/LTQOrbitrap/MS/MSn analysis.

N°	Rt	Molecular Formula	Identity	EB	ET
**1**	1.81	C_12_ H_20_ O_10_	agarobiose	x	x
**2**	4.78	C_7_ H_10_ O_7_	citric acid methyl ester	x	
**3**	5.54	C_9_ H_17_ O_5_ N	pantothenic acid		x
**4**	6.8	C_34_ H_24_ O_22_	pedunculagin	x	
**5**	7.17	C_41_ H_28_ O_27_	geranin	x	
**6**	7.22	C_16_ H_18_ O_9_	chlorogenic acid	x	
**7**	7.4	C_27_ H_31_ O_16_^+^	cyanidin dihexoside	x	
**8**	7.72	C_27_ H_22_ O_18_	galloyl HHDP glucose	x	
**9**	7.81	C_30_H_26_O_12_	EC-b-EC		x
**10**	7.94	C_22_ H_22_ O_13_	laricitrin glucoside	x	
**11**	7.98	C_21_ H_21_ O_11_^+^	cyanidin 3-*O*-glucoside	x	
**12**	8.56	C_21_ H_21_ O_10_^+^	pelargonidin 3-*O*-glucoside	x	
**13**	8.86	C_20_H_27_NO_11_	amygdalin		x
**14**	8.86	C_15_H_14_O_6_	(+)-catechin		x
**15**	8.86	C_45_H_38_O_18_	EC-b-EC-b-EC		x
**16**	8.88	C_21_ H_22_ O_12_	dihydroquercetin glucoside	x	x
**17**	8.93	C_20_ H_19_ O_10_^+^	cyanidin xyloside	x	
**18**	9.13	C_24_ H_22_ O_14_	kaempferol malonyl glucoside	x	
**19**	9.27	C_30_H_27_O_12_	EC-b-EC		x
**20**	9.37	C_20_ H_20_ O_11_	dihydroquercetin pentoside	x	
**21**	9.42	C_27_ H_28_ O_15_	cyanidin dioxalyl glucoside	x	
**22**	9.83	C_15_H_14_O_6_	(-)-epicatechin		x
**23**	10.16	C_45_H_38_O_18_	EC-b-EC-b-EC		x
**24**	10.25	C_45_H_36_O_18_	EC-b-EC-a-EC		x
**25**	10.52	C_41_ H_26_ O_26_	castalagin	x	
**26**	10.74	C_20_ H_16_ O_12_	quercetin 3-*O*- rhamnoside	x	x
**27**	10.81	C_21_ H_16_ O_14_	methyl ellagic acid glucuronide	x	x
**28**	11.43	C_30_H_24_O_12_	EC-a-EC		x
**29**	11.47	C_41_ H_28_ O_26_	galloyl bis HHDP glucose	x	
**30**	11.52	C_21_ H_21_ O_12_^+^	delphinidin 3-*O*-glucoside	x	
**31**	11.59	C_21_ H_22_ O_11_	eriodictyol-7-*O*-glucoside		x
**32**	11.61	C_21_ H_20_ O_12_	quercetin 3-*O*-glucoside	x	x
**33**	11.68	C_21_ H_20_ O_11_	kaempferol 3-*O*-glucoside	x	x
**34**	11.77	C_21_ H_18_ O_13_	quercetin glucuronide	x	
**35**	11.81	C_28_H_32_O_16_	isorhamnetin rutinoside		x
**36**	11.85	C_27_H_30_O_15_	kaempferol 3-*O*-rutinoside	x	x
**37**	11.95	C_26_ H_34_ O_11_	icariside E5		x
**38**	12.01	C_27_ H_18_ O_16_	quercetin hydroxy-methylglutaroy-gluc	x	
**39**	12.48	C_22_H_22_O_12_	isorhamnetin glucoside	x	x
**40**	12.92	C_21_ H_22_ O_10_	prunin		x
**41**	13.02	C_22_ H_20_ O_13_	isorhamnetin glucuronide		x
**42**	13.02	C_21_ H_20_ O_12_	iperoside	x	x
**43**	13.06	C_37_ H_60_ O_13_	unknown	x	
**44**	13.06	C_42_ H_54_ O_9_	unknown	x	
**45**	13.21	C_30_ H_38_ O_10_	sesquimarocanol B		x
**46**	13.41	C_36_ H_56_ O_12_	suavissimoside f 1	x	
**47**	13.68	C_18_ H_16_ O_8_	rosmarinic acid		x
**48**	13.91	C_20_ H_22_ O_8_	piceid		x
**49**	15.26	C_30_ H_18_ O_14_	unknown	x	
**50**	15.88	C_18_ H_32_ O_5_	9,12,13-TriHODE (10, 15)		x
**51**	16.16	C_72_ H_110_ O_24_	coreanoside F1	x	
**52**	16.27	C_21_ H_40_ O_7_	unknown		x
**53**	16.55	C_15_ H_10_ O_7_	quercetin	x	
**54**	17.54	C_30_ H_46_ O_8_	trachelosperogenin C	x	
**55**	17.77	C_18_ H_34_ O_5_	9,12,13-TriHOME (10)		x
**56**	18.56	C_30_ H_48_ O_6_	19α-hydroxyasiatic acid	x	
**57**	20.3	C_30_ H_46_ O_7_	corosin	x	
**58**	26.02	C_60_ H_90_ O_14_	coreanogenoic acid	x	
**59**	17.33	C_30_ H_46_ O_6_	3-epiilexgenin A	x	

**Table 3:** Secondary metabolites quantified by UHPLC coupled to Qtrap 6500 (AbSciex).

	EB	ET	EM	AB	AM	Sign.
Anthocyanins						
**11**	2685.00 ± 42.43	nd	548.87 ± 41.43	4467.50 ± 236.88	1975.90 ± 107.38	**
**12**	2671.05 ± 43.78	nd	77.68 ± 2.77	3766.25 ± 290.06	88.04 ± 7.50	**
**17**	651.56 ± 22.09	nd	158.47 ± 31.40	948.44 ± 71.82	499.30 ± 115.04	**
**21**	54.84 ± 3.09	nd	nd	62.54 ± 0.16	nd	**
**30**	1780.00 ± 120.21	nd	383.25 ± 0.35	3762.50 ± 434.87	2907.50 ± 152.03	
Flavanols						
**14**	nd	6.47 ± 0.01	nd	nd	nd	**
**19**	nd	23.60 ± 3.39	nd	nd	nd	**
**22**	nd	5.06 ± 0.51	nd	nd	nd	**
Flavonols						
**26**	232.70 ± 1.15	0.91 ± 0.02	211.45 ± 0.47	265.44 ± 16.25	222.22 ± 2.74	**
**32**	451.24 ± 21.34	1.68 ± 0.60	260.48 ± 5.09	720.20 ± 1.64	424.15 ± 10.95	**
**33**	1842.50 ± 222.74	27.59 ± 21.2	915.00 ± 141.42	3442.50 ± 1686.45	1667.50 ± 258.09	**
**36**	1850.00 ± 45.96	7.36 ± 0.14	1835.00 ± 14.14	1837.50 ± 3.53	1872.50 ± 53.03	**
**53**	240.75 ± 10.25	0.91 ± 0.01	209.25 ± 0.35	262.50 ± 4.24	221.25 ± 1.06	**
Other compounds						
**6**	205.00 ± 10.05	nd	nd	390.00 ± 14.14	nd	**
**47**	nd	23.92 ± 9.40	nd	nd	nd	
**40**	nd	1.91 ± 0.97	nd	nd	nd	

EB = ethanolic extract of blackberry fruits; ET = ethanolic extract of almond skin; EM = ethanolic extract of blackberry jam; AB = anthocyanin extract of blackberry fruits; AM = anthocyanin extract of blackberry jam. Results are given as means ± standard deviation (SD). nd: not detected. Concentrations are reported as µg/g DW; anthocyanins [cyanidin 3-*O*-glucoside (**11**), pelargonidin 3-*O*-glucoside (**12**), cyanidin xyloside (**17**), cyanidin dioxayl glucoside (**21**), delphinidin 3-*O*-glucoside (**30**)], flavanols [(+)-catechin (**14**), proanthocyanidin B (**19**), and (-)-epicatechin (**22**)], and flavonols [quercetin 3-*O*-rhamnoside (**26**), quercetin 3-*O*-glucoside (**32**), kaempferol 3-*O*-glucoside (**33**), kaempferol 3-*O*-rutinoside (**36**), quercetin (**53**)], chlorogenic acid (**6**), rosmarinic acid (**47**), and prunin (**40**)]. Means within a row with different letters are significantly different by Tukey’s post hoc test. ** Significance at *p* < 0.01.

**Table 4:** GC–MS analysis of almond skin *n*-hexane extract.

R_t_ (min)	Fatty Acid	%
15.78	Myristic acid	0.43 ± 0.01
16.55	Pentadecanoic acid	0.46 ± 0.02
17.16	Palmitoleic acid	3.40 ± 0.24
17.31	Palmitic acid	16.65 ± 1.32
18.00	Margaric acid	0.37 ± 0.02
18.41	Methyl γ-linolenate	1.68 ± 2.14
18.56	Linoleic acid	63.19 ± 2.56
18.63	Oleic acid	8.41 ± 0.21
18.71	Stearic acid	4.10 ± 0.14
19.95	Arachidic acid	0.72 ± 0.13
21.14	Behenic acid	0.54 ± 0.21
	Total identified	99.95

Data are reported as mean ± standard deviation (*n* = 3).

**Table 5:** Antioxidant activity of investigated samples.

Extract	DPPH Test IC_50_ (µg/mL)	ABTS Test IC_50_ (µg/mL)	FRAP Test μMFe (II)/g	β-Carotene Bleaching Test IC_50_ (µg/mL)	
				t = 30 min	t = 60 min
Blackberry fruit					
EB	38.03 ± 2.11 ***	11.81 ± 1.28 ***	87.47 ± 4.17 ^ns^	15.10 ± 1.51 ****	16.38 ± 1.64 ****
AB	1.48 ± 0.12 ^ns^	0.21 ± 0.07 ^ns^	102.39 ± 5.32 ***	2.15 ± 0.31 *	3.92 ± 0.53 **
Blackberry jam					
EM	47.84 ± 6.59 ****	63.81 ± 3.64 ****	18.30 ± 1.83 ****	53.97 ± 3.08 ****	59.78 ± 3.21 ****
AM	1.86 ± 0.27 ^ns^	2.21 ± 0.45 ^ns^	92.71 ± 4.54 ^ns^	3.34 ± 0.51 *	4.51 ± 0.62 **
Almond skin					
ET	35.40 ± 2.05 ***	2.54 ± 0.44 **	21.38 ± 1.74 ****	9.09 ± 1.18 ***	13.19 ± 1.41 ****
ES	78.45 ± 3.82 ***	49.42 ± 2.95 ***	4.84 ± 0.8 ****	53.22 ± 2.0 ****	61.03 ± 2.2 ****
Positive control					
Ascorbic acid	5.01 ± 0.81	1.72 ± 0.06			
BHT			63.22 ± 2.34		
Propyl gallate				0.09 ± 0.04	0.09 ± 0.04

EB: ethanol/H_2_O blackberry extracts; AB: anthocyanin blackberry extracts; EM: ethanol/H_2_O blackberry jam extracts; AM: anthocyanin blackberry jam extracts; ET: almond skin ethanolic extract; ES: almond skin *n*-hexane extract. Data are expressed as means ± S.D. (*n* = 3). Differences within and between groups were evaluated by one-way ANOVA followed by a multi-comparison Dunnett’s test (α = 0.05): **** *p* < 0.0001. *** *p* < 0.001. ** p < 0.01. * *p* < 0.1 compared with the positive controls. ns: not significant.

**Table 6:** Hypoglycaemic and hypolipidemic activity [IC_50_ (µg/mL)] of investigated samples.

Extract	α-Amylase	α-Glucosidase	Lipase
Blackberry fruit			
EB	113.17 ± 5.11 ****	55.48 ± 3.14 **	67.98 ± 3.67 ****
AB	17.59 ± 1.44 ^ns^	12.77 ± 1.23 ^ns^	23.37 ± 1.91 ^ns^
Blackberry jam			
EM	108.24 ± 4.89 ****	125.65 ± 5.04 ****	97.86 ± 4.45 ****
AM	28.42 ± 2.03 ^ns^	15.75 ± 1.28 ^ns^	30.16 ± 2.14 ^ns^
Almond skin			
ET	73.76 ± 3.89 ****	57.09 ± 2.57 **	49.35 ± 2.14 ***
ES			
Positive control			
Acarbose	35.50 ± 0.93	50.11 ± 1.31	
Orlistat			37.42 ± 1.08

EB: ethanol/H_2_O blackberry extracts; AB: anthocyanin blackberry extracts; EM: ethanol/H_2_O blackberry jam extracts; AM: anthocyanin blackberry jam extracts; ET: almond skin ethanolic extract; ES: almond skin *n*-hexane extract. Data are expressed as means ± S.D. (*n* = 3). Acarbose and orlistat were used as positive control. Differences within and between groups were evaluated by one-way ANOVA, followed by a multi-comparison Dunnett’s test (α = 0.05): **** *p* < 0.0001, *** *p* < 0.001, ** p < 0.01 compared with the positive controls. ns: Not significant.

**Table 7:** Antioxidant activity (%) of enriched and pasteurised enriched jam.

	DPPH Test	ABTS Test	FRAP Test	β-Carotene Bleaching Test	
				t = 30 min	t = 60 min
Enriched jam					
EMT1	85.39 ± 2.12 ****	92.93 ± 2.54 ****	62.86 ± 1.27 ****	89.79 ± 2.28 ****	83.02 ± 2.01 ****
EMT2	79.47 ± 1.97 ****	81.56 ± 2.08 ****	59.71 ± 1.20 **	82.24 ± 2.17 ****	79.88 ± 1.97 ****
EMT3	71.46 ± 1.56 ***	78.08 ± 1.81 **	56.52 ± 1.18 *	80.66 ± 2.09 ****	78.97 ± 1.95 ****
Pasteurised enriched jam					
EMTP1	74.46 ± 2.89 ****	80.12 ± 2.92 ****	51.78 ± 1.87 ****	80.49 ± 2.94 ****	75.48 ± 2.36 ****
EMTP2	67.10 ± 2.57 ****	78.46 ± 2.96 ****	50.04 ± 1.76 ****	72.85 ± 2.26 ****	69.74 ± 2.67 ****
EMTP3	60.45 ± 2.10 **	72.96 ± 2.24 ****	47.21 ± 1.42 **	69.78 ± 2.64 ****	62.31 ± 2.51 ****
Negative control					
EM	65.25 ± 1.02	71.45 ± 1.27	54.61 ± 1.01	63.46 ± 1.06	62.36 ± 1.07

EM: jam without almond skin; EMT1: enriched jam with 20% of T; EMT2: enriched jam with 15% of T; EMT3: enriched jam with 10% of T; EMP: jam without almond skin; EMTP1: enriched jam with 20% of T; EMTP2: enriched jam with 15% of T; EMTP3: enriched jam with 10% of T. Data are expressed as means ± S.D. (*n* = 3). DPPH radical scavenging activity assay; antioxidant capacity determined by radical cation (ABTS+); ferric reducing antioxidant power (FRAP). Ascorbic acid. BHT and propyl gallate were used as a positive control in antioxidant tests. Differences within and between groups were evaluated by one-way ANOVA followed by a multi-comparison Dunnett’s test (α = 0.05): **** *p* < 0.0001, *** *p* < 0.001, ** p < 0.01, * p < 0.1, compared with the negative controls (EM).

**Table 8:** Hypoglycaemic and hypolipidemic activity (%) of jam.

Sample	α-Amylase	α-Glucosidase	Lipase
Enriched jam			
EMT1	91.23 ± 3.25 ****	103.56 ± 3.58 ****	96.74 ± 3.58 ****
EMT2	86.94 ± 2.87 ****	92.79 ± 3.42 ****	91.74 ± 3.24 ****
EMT3	85.18 ± 2.47 ****	87.46 ± 2.92 **	88.76 ± 2.97 ****
Pasteurised enriched jam			
EMTP1	84.96 ± 3.01 ****	89.78 ± 3.25 ****	86.97 ± 3.17 ****
EMTP2	80.12 ± 2.85 ****	86.41 ± 3.18 ****	82.48 ± 3.12 ****
EMTP3	76.37 ± 2.74 ****	80.14 ± 2.86 ***	80.16 ± 2.95 ****
Negative control			
EMP	65.57 ± 2.45	71.58 ± 2.67	63.78 ± 2.39

EM: jam without almond skin; EMT1: enriched jam with 20% of T; EMT2: enriched jam with 15% of T; EMT3: enriched jam with 10% of T; EMP: jam without almond skin; EMTP1: enriched jam with 20% of T; EMTP2: enriched jam with 15% of T; EMTP3: enriched jam with 10% of T. Differences within and between groups were evaluated by one-way ANOVA followed by a multi-comparison Dunnett’s test (α = 0.05): **** *p* < 0.0001; *** *p* < 0.001; ** *p* < 0.01 compared with the negative control.

## Data Availability

Data is contained within the article and Supplementary Materials.

## Supplementary Materials

The following are available online at https://www.mdpi.com/article/10.3390/antiox10081218/s1, Table S1. Total phytochemical contents in blackberry fruits, jam, and almond skin extract. Table S2. Secondary metabolites identified in ethanol extract of blackberry fruits (EB) and in ethanol extract of almond skin (ET) by LC-ESI/LTQOrbitrap/MS/MSn analysis, operating in positive and negative ion mode. Table S3. Sensory analysis of pasteurised enriched jam. Figure S1. Phytochemicals content in enriched jam (a) and pasteurised enriched jam (b). Figure S2. Sensory profile of enriched jams.
