# Supplementary material for: Almond (Prunus dulcis cv. Casteltermini) Skin Confectionery By-Products: New Opportunity for the Development of a Functional Blackberry (Rubus ulmifolius Schott) Jam
Source: Antioxidants (Basel). 2021 Jul 29;10(8):1218. doi: 10.3390/antiox10081218 (PMC8388876; doi:10.3390/antiox10081218)
Supplement: Supplementary file 1 [file antioxidants-10-01218-s001.zip › antioxidants-1293273-supplementary.pdf]

Supplementary materials

Table S1. Total phytochemical contents in blackberries fruits, jam and almond skin extract

Samples	TPC (mg/g extract)	TFC (mg/g extract)	TAC (mg/g extract)
**Blackberries extracts**			
EB	189.31 ± 5.32[a]	30.26 ± 2.92[a]	1.13 ± 0.32[c]
AB	-	-	7.52 ± 0.98[a]
**Blackberries jam extracts**			
EM	20.57 ± 2.85[c]	18.98 ± 2.34[c]	0.60 ± 0.18[d]
AM	-	-	2.06 ± 0.68[b]
**Almond skin extract**			
ET	56.68 ± 3.12[b]	20.38 ± 2.72[b]	NT
**Sign.**	**	**	**

EB: Ethanol/H_2O blackberries extracts; AB: Anthocyanin blackberries extracts; EM: Ethanol/H_2O blackberries jam extracts; AM: Anthocyanin blackberries jam extracts; ET: almond seed coat ethanolic extract; ES: almond seed coat *n*-hexane extract. TPC: Total phenolsNT: not tested. Data are expressed as mean ± S.D. (n= 3). Differences were evaluated by one-way analysis of variance (ANOVA) completed with a multicomparison Tukey's test. ** $p < 0.05$. Means in the same column with different small letters differ significantly ($p < 0.05$). Sign: significant.

Table S2. Secondary metabolites identified in ethanol extract of blackberries fruits (EB) and in ethanol extract of almond seed coat (ET) by LC-ESI/LTQOrbitrap/MS/MSn analysis, operating in positive and negative ion mode.

N°	Rt	$[M+H]^+$	$[M-H]^-$	Molecular Formula	ppm	MS/MS	Identity	EB	ET
**1**	1.81	325.1116		$C_{12}H_{20}O_{10}$	-3.88	271.08/289.09/127.03	agarobiose	x	x
**2**	4.78		205.0350	$C_{7}H_{10}O_{7}$	3.80	173.01	citric acid methyl ester	x	
**3**	5.54		218.1031	$C_{9}H_{17}O_{5}N$	3.35	88.04/146.08	pantothenic acid		x
**4**	6.8		783.0677	$C_{34}H_{24}O_{22}$	0.25	481.06/301.00	pedunculagin	x	
**5**	7.17		951.0728	$C_{41}H_{28}O_{27}$	-0.64	469.00/783.07/907.08	geranin	x	
**6**	7.22	355.1012		$C_{16}H_{18}O_{9}$	-3.17	163.04	chlorogenic acid*	x	
**7**	7.4	611.1591		$C_{27}H_{31}O_{16}$	-2.52	449.11/287.05	cyanidin dihexoside		
**8**	7.72		633.0715	$C_{27}H_{22}O_{18}$	-1.12	301	galloyl HHDP glucose	x	
**9**	7.81		577.1337	$C_{30}H_{26}O_{12}$	0.57	559/451/ 425/407/ 289	EC-b-EC		x
**10**	7.94		493.0979	$C_{22}H_{22}O_{13}$	0.47	269.04/449.11/313.03	laricitrin glucoside	x	
**11**	7.98	449.1059		$C_{21}H_{21}O_{11}$	-4.30	287.05	cyanidin 3-*O*-glucoside*	x	
**12**	8.56	433.1117		$C_{21}H_{21}O_{10}$	-2.68	271.06	pelargonidin 3-*O*-glucoside*	x	
**13**	8.86		456.1498	$C_{20}H_{27}NO_{11}$	0.84	323/263/221/179	amygdalin		x
**14**	8.86		289.0709	$C_{15}H_{14}O_{6}$	1.02	245/205/179	(+)-catechin*		x
**15**	8.86		865.1960	$C_{45}H_{38}O_{18}$	0.95	739/713/695/577/ 449/ 407	EC-b-EC-b-EC		x
**16**	8.88		465.1025	$C_{21}H_{22}O_{12}$	-0.39	285.04/303.05/241.05	dihydroquercetin glucoside	x	x
**17**	8.93	419.0962		$C_{20}H_{19}O_{10}$	-2.44	287.05	cyanidin xyloside*	x	
**18**	9.13	535.1059		$C_{24}H_{22}O_{14}$	-4.35	287.05	kaempferol malonyl glucoside	x	
**19**	9.27		577.1337	$C_{30}H_{27}O_{12}$	0.63	559/ 451/425/407/ 289	EC-b-EC		x
**20**	9.37	437.1066		$C_{20}H_{20}O_{11}$	-2.76	305.06/419.09	dihydroquercetin pentoside	x	
**21**	9.42	593.1481		$C_{27}H_{28}O_{15}$	-3.31	287.05	cyanidin dioxayl glucoside	x	
**22**	9.83		289.0707	$C_{15}H_{14}O_{6}$	1.58	245/205/ 179	(-)-epicatechin*		x
**23**	10.16		865.1963	$C_{45}H_{38}O_{18}$	1.61	739/ 713/ 695/ 577/575/449/407/ 287	EC-b-EC-b-EC		x
**24**	10.25		863.1806	$C_{45}H_{36}O_{18}$	1.19	711/693/ 575/573	EC-b-EC-a-EC		x
**25**	10.52		933.0624	$C_{41}H_{26}O_{26}$	-0.44	633.07/301.00	castalagin	x	
**26**	10.74		447.0553	$C_{20}H_{16}O_{12}$	-0.50	299.99/301.00	quercetin 3-*O*- rhamnoside	x	x
**27**	10.81		491.0454	$C_{21}H_{16}O_{14}$	-0.19	315.01	methyl ellagic acid glucuronide	x	x
**28**	11.43		575.1182	$C_{30}H_{24}O_{12}$	0.44	539/ 529/ 449/ 423/407/ 289	EC-a-EC		x
**29**	11.47		935.0773	$C_{41}H_{28}O_{26}$	-1.25	633.07/301.00	galloyl bis HHDP glucose	x	
**30**	11.52	465.1016		$C_{21}H_{21}O_{12}$	-2.43	303.05	delphinidin 3-*O*-glucoside*	x	
**31**	11.59		449.1083	$C_{21}H_{22}O_{11}$	1.11	287	eriodictyol-7-O-glucoside		x
**32**	11.61		463.0868	$C_{21}H_{20}O_{12}$	-0.25	301	quercetin 3-*O*-glucoside*	x	x
**33**	11.68		447.0923	$C_{21}H_{20}O_{11}$	0.16	285.01	kaempferol 3-*O*-glucoside*	x	x
**34**	11.77	479.0798		$C_{21}H_{18}O_{13}$	-4.50	303.05	quercetin glucuronide	x	
**35**	11.81		623.1607	$C_{28}H_{32}O_{16}$	0.64	315/ 300	isorhamnetin rutinoside		x
**36**	11.85		593.1490	$C_{27}H_{30}O_{15}$	0.54	285	kaempferol 3-*O*-rutinoside*	x	x
**37**	11.95		521.2027	$C_{26}H_{34}O_{11}$	1.84	359.15	Icariside E5		x
**38**	12.01	609.1431		$C_{27}H_{18}O_{16}$	-3.08	303.03/345.05/591.13	quercetin hydroxy-methylglutaroy-gluc	x	
**39**	12.48		477.1023	$C_{22}H_{22}O_{12}$	1.68	357/315	isorhamnetin hexoside	x	x
**40**	12.92		433.1128	$C_{21}H_{22}O_{10}$	0.16	271.06	prunin*		x
**41**	13.02	493.0965		$C_{22}H_{20}O_{13}$	-2.36	303.05	isoramnetin glucuronide		
**42**	13.02	465.1010		$C_{21}H_{20}O_{12}$	-3.68	303.05	iperoside	x	x
**43**	13.06		711.3944	$C_{37}H_{60}O_{13}$	-0.77	503.34/665.39	unknown	x	
**44**	13.06		701.3658	$C_{42}H_{54}O_{9}$	-3.66	503.34	unknown	x	
**45**	13.21		557.2390	$C_{30}H_{38}O_{10}$	1.59	509.22/539.23/415.18/361.17	sesquimarocanol B		x
**46**	13.41		679.3677	$C_{36}H_{56}O_{12}$	1.56	517.31/499.30/283.85	suavissimoside f 1	x	
**47**	13.68		359.0768	$C_{18}H_{16}O_{8}$	0.69	161.02/197.05/179.03	rosmarinic acid*		x
**48**	13.91		389.1235	$C_{20}H_{22}O_{8}$	0.40	359.11/179.04	piceid		x
**49**	15.26		601.0604	$C_{30}H_{18}O_{14}$	-1.43	465.05/437.05/301.03	unknown	x	
**50**	15.88		327.2174	$C_{18}H_{32}O_{5}$	2.65	309.21/291.20/269.18/171.10	9,12,13-TriHODE (10,15)		x
**51**	16.16		1357.7259	$C_{72}H_{110}O_{24}$	-2.86		coreanoside F1	x	

**52**	16.27		403.2698	$C_{21}H_{40}O_7$		311.22/329.23	unknown		x
**53**	16.55	303.0490		$C_{15}H_{10}O_7$	-2.83	257.04/285.03/229.04/165.01	quercetin	x	
**54**	17.54		533.3110	$C_{30}H_{46}O_8$	0.27	485.29/471.31/283.85	trachelosperogenin C	x	
**55**	17.77		329.2328	$C_{18}H_{34}O_5$	0.62	229.14/211.13/171.10	9,12,13-TriHOME (10)		x
**56**	18.56		503.3366	$C_{30}H_{48}O_6$	-0.21	485.33/441.34/453.30	19α-hydroxyasiatic acid	x	
**57**	20.3		517.3158	$C_{30}H_{46}O_7$	-0.23	437.31/455.32	corosin	x	
**58**	26.02		1033.6231	$C_{60}H_{90}O_{14}$	-1.46	981.30/283.85	coreanogenoic acid	x	
**59**	17.33		501.3207	$C_{30}H_{46}O_6$	-0.70	483.31/421.31/439.32/403.30	3-epiilexgenin A	x	

*Compounds identified with standards

Table S3. Sensory analysis of pasteurized enriched jam

Samples	Appearance	Colour	Odour	Aroma	Sweetness	Acidity	Mouthfeel
EMP	7.98^{a}	7.82^{a}	8.02^{d}	7.84^{b}	8.42^{a}	7.95^{a}	7.75^{a}
EMTP1	8.01^{a}	7.83^{a}	8.42^{a}	8.56^{a}	7.94^{b}	7.96^{a}	7.43^{b}
EMTP2	8.02^{a}	7.82^{a}	8.41^{b}	8.56^{a}	7.92^{b}	7.95^{a}	7.42^{b}
EMTP3	8.01^{a}	7.83^{a}	8.38^{c}	8.54^{a}	7.92^{b}	7.94^{a}	7.42^{b}
Sign.	ns	ns	**	**	**	ns	**

Data are expressed as mean ± S.D. (n= 3). EMP: jam without almond skin; EMTP1: enriched jam with 20% of T; EMTP2: enriched jam with 15% of T; EMTP3: enriched jam with 10% of T. Differences were evaluated by one-way analysis of variance (ANOVA) completed with a multicomparison Tukey's test. ** $p < 0.05$. Means in the same column with different small letters differ significantly ($p < 0.05$). Sign: significant.

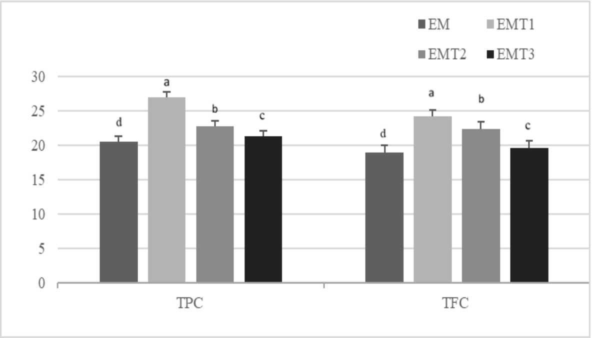

(a)

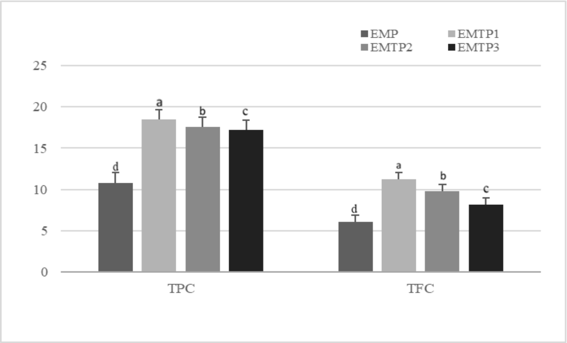

(b)

Figure S1. Phytochemicals content in enriched jam (**a**) and pasteurized enriched jam (**b**). Data are expressed as mean ± S.D. ($n = 3$). TPC: Total phenol content expressed as mg of chlorogenic acid equivalents (CAE)/g of extract. TFC: total flavonoid content expressed as mg quercetin equivalents (QE)/g of extract. Differences were evaluated by one-way analysis of variance (ANOVA) completed with a multicomparison Tukey's test. Means in the same column with different small letters differ significantly ($p < 0.05$).

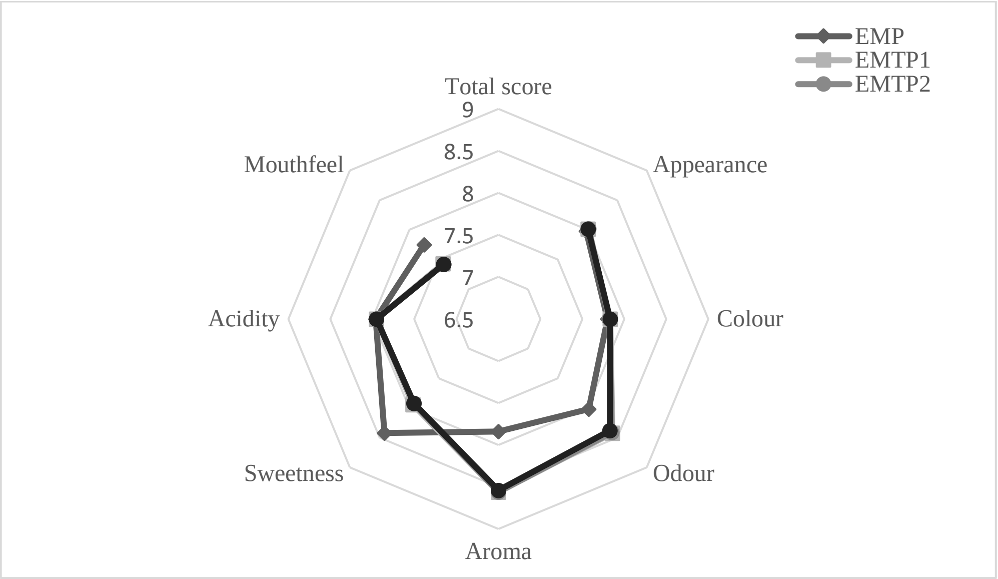

Figure 2. Sensory profile of enriched jams.
